# Sleep in adolescents of different socioeconomic status: a systematic review

**DOI:** 10.1016/j.rpped.2015.01.011

**Published:** 2015

**Authors:** Érico Pereira Gomes Felden, Carina Raffs Leite, Cleber Fernando Rebelatto, Rubian Diego Andrade, Thais Silva Beltrame

**Affiliations:** a Universidade do Estado de Santa Catarina (UDESC), Florianópolis, SC, Brazil

**Keywords:** Sleep, Adolescent, Social class

## Abstract

**Objective:**

To analyze the sleep characteristics in adolescents from different socioeconomic levels.

**Data source:**

Original studies found in the MEDLINE/PubMed and SciELO databases without language and period restrictions that analyzed associations between sleep variables and socioeconomic indicators. The initial search resulted in 99 articles. After reading the titles and abstracts and following inclusion and exclusion criteria, 12 articles with outcomes that included associations between sleep variables (disorders, duration, quality) and socioeconomic status (ethnicity, family income, and social status) were analyzed.

**Data synthesis:**

The studies associating sleep with socioeconomic variables are recent, published mainly after the year 2000. Half of the selected studies were performed with young Americans, and only one with Brazilian adolescents. Regarding ethnic differences, the studies do not have uniform conclusions. The main associations found were between sleep variables and family income or parental educational level, showing a trend among poor, low social status adolescents to manifest low duration, poor quality of sleeping patterns.

**Conclusions:**

The study found an association between socioeconomic indicators and quality of sleep in adolescents. Low socioeconomic status reflects a worse subjective perception of sleep quality, shorter duration, and greater daytime sleepiness. Considering the influence of sleep on physical and cognitive development and on the learning capacity of young individuals, the literature on the subject is scarce. There is a need for further research on sleep in different realities of the Brazilian population.

## Introduction

People go through important changes during the course of their lives, both in terms of physical shape and behavior. In adolescence, in particular, one can observe important changes in the sleep/wake cycle, including a delay in the sleep phase, characterized by later bed- and wake-up times.^1^^,^ 2 This biological tendency of adolescents can be accentuated by behaviors such as the use of computers, games and TV at night. Additionally, environmental issues, such as social commitments early in the morning, increase the prevalence of short sleep duration in this population.^3^

The study by Bernardo et al.^4^ identified a prevalence of 39% of adolescents with short sleep duration in São Paulo. Perez-Chada et al.^5^ observed that 49% of the assessed Argentinean adolescents had short sleep duration. Sleep disorders have been associated with several health outcomes, such as cognitive development disorders,^6^ psychiatric disorders,^7^ metabolic and excess weight disorders,^8^^,^ 9 as well as a higher degree of stress.^10^

In addition to the biological issues, the environment seems to have a decisive influence on the sleep/wake cycle. In this context, the literature indicates that socioeconomic status is one of the most relevant social variables for the understanding of health issues.^11^^-^ 13 As for sleep, the studies are scarce and this association is little explored, especially regarding studies with adolescents. However, it is known that acknowledging the associations and causal links between sleep and socioeconomic status is fundamental for the understanding of adolescent sleep and to mediate a proposal for health education.

Considering the abovementioned facts and taking into account the importance of studies that investigate the association between sleep and socioeconomic status for the planning of public health actions and the scarcity of studies that summarize the literature on this topic, this study aimed to make a systematic review to evaluate the association between sleep characteristics in adolescents from different socioeconomic levels.

## Method

**Figure 1:**
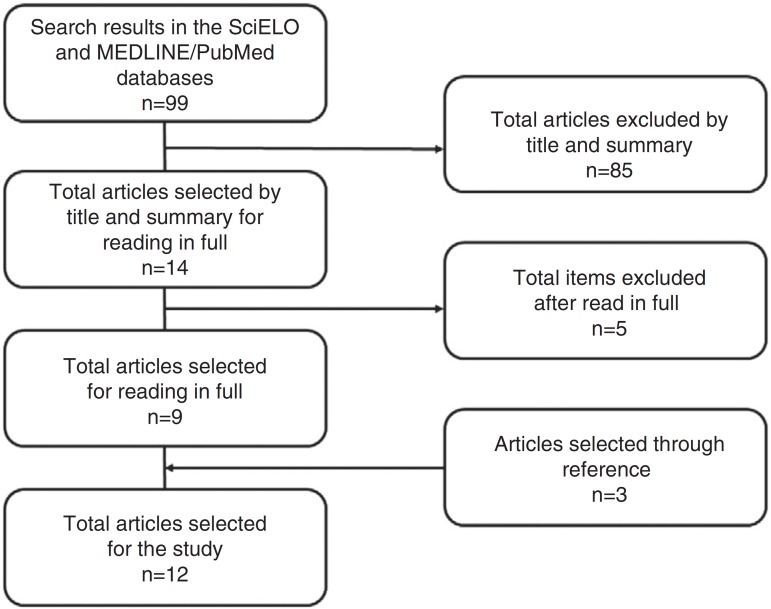
Flow chart of the article selection process for the review.

A systematic literature review was performed using the SciELO and MEDLINE/PubMed databases, with no period limitations or language exclusion. The search used the terms "sleep" and "socioeconomic status" together with the term "adolescents," as well as the equivalent terms in Portuguese. Additionally, the search was expanded by analyzing the relevant studies found in the references of articles selected in the initial search. The first search resulted in a total of 99 studies, as described in Fig. 1.

Based on the initial search, the articles selected for analysis had to meet the following inclusion criteria: (a) original articles with sleep variable results (duration and quality of sleep, sleep efficiency and mild sleep disorders, such as insomnia); (b) studies with adolescent samples; and (c) articles that showed measures of association and/or differences between the sleep variables and socioeconomic indicators. Articles limited to populations with specific conditions, such as mental retardation and heart disease were excluded from this review.

Considering the abovementioned inclusion and exclusion criteria, based on the reading of titles and abstracts, 85 articles were excluded as they did not stratify adolescents from children's and adult's samples, did not include adolescents in the assessed population and/or only analyzed populations with chronic, noncommunicable diseases and/or with more severe sleep disorders. Additionally, the main cause of exclusion was the fact that some articles showed sleep and socioeconomic status as independent variables, not showing associations or differences between them. Therefore, we selected 14 studies to be read in full. After that, the articles were read; the information was extracted from the full texts and discussed in a group, resulting in the selection of nine studies. Additionally, we included three more articles found in the references, totaling 12 articles included in the final analysis. The data extraction was performed independently by four investigators, whereas the analysis and results were discussed in a group.

Article quality assessment was performed using the proposal created by Downs and Black,^14^ consisting of 27 questions that estimate communication, external validity, internal validity (bias and confounding factors) and statistical power. This assessment was performed by two authors and, in cases of doubt, a third reviewer was consulted for a final decision. For the present study, questions 8, 13, 14, 15, 17, 19, 20, 21, 22, 23, 24 and 26 were excluded, as intervention studies were not included in the review, and thus a total of 15 questions were analyzed. According to the quality assessment proposal, the questions received a score of zero (0) or one (1) point, except for question five, which ranged from zero (0) to two (2) points. Moreover, question 27, which analyzes statistical power, varied from zero (0) to five (5) points. Thus, an article could attain a maximum score of 20 points. Given the low number of selected articles, this analysis aimed to discuss factors related to article quality and was not an exclusion criterion.

## Results

Tables 1 and 2 disclose general information about the 12 studies included in this review, according to the date of publication (2000-2009 in Table 1 and 2010 onwards in Table 2). The samples come from different countries: six from the USA, one from New Zealand, one from Australia, one from Greece, one from Norway, one from Canada and one from Brazil. Article publication occurred from 2000 to 2013; 11 of them had a cross-sectional design and only one was a longitudinal study.

**Table 1:** Synthesis of the selected studies published between 2000 and 2009.

Authors	Sample/nationality	Sleep variables	Socioeconomic variables	Results	D&B score
Roberts et al. (2000)^15^	5423 individuals from the USA aged 10–17 years	Insomnia and hypersomnia	Ethnicity and perception of socioeconomic status	Adolescents from different ethnic backgrounds showed differences related to insomnia and hypersomnia. Insomnia was more frequent in groups of African and Hispanic descent. Adolescents of Chinese descent were less likely to have sleep problems.	13
Roberts et al. (2004)^16^	5118 individuals from the USA aged 13–18 years	Insomnia, sleep duration, quality of sleep and restful sleep	Ethnicity and family life style	The study showed significant differences between ethnic groups regarding insomnia and sleep quality. Insomnia was more prevalent among poor adolescents. The associations between insomnia and ethnicity were not significant after adjusting for confounders. The adolescents classified as “poor” were 4.5 times more likely to report insomnia.	12
Roberts et al. (2006)^17^	4.175 individuals from the USA aged 11 to 17 years and adult caregivers	Problems falling asleep, nighttime awakenings, insomnia and unrestful sleep	Ethnicity, family income and level of schooling of caregivers	Young individuals with low family income were more likely to develop sleep disorders. Individuals of European descent were more likely to have trouble sleeping, have sleeping problems (such waking up during the night) and difficulty falling asleep than those of African descent. Young individuals of Hispanic descent had a lower prevalence of sleep problems than the ones of European descent.	11
Smaldon et al. (2007)^18^	68,418 individuals from the USA aged 6 to 17 years	Quality of sleep	Ethnicity, parental level of schooling, income and skin color	Non-Hispanic white adolescents, with higher levels of family education, were approximately 30% more likely to have inadequate sleep patterns. However, it was verified that adolescents with lower family income were 50% less likely to have inadequate sleep patterns, having as reference the adolescents with higher family income.	14
Dollman et al. (2007)^6^	900 individuals from Australia aged 10–15 years (390 in 1985 and 510 in 2004)	Duration of sleep, bedtime and wake up time	Australian socioeconomic index	When comparing the two evaluations, there was a significant reduction in sleep duration in young individuals with lower socioeconomic status (44 min) than in those with higher socioeconomic status.	9
Bernardo et al. (2009)^4^	863 individuals from Brazil aged 10–19 years	Sleep duration and sleep disorders	Social classes	Sleep duration showed decreasing trend with increasing socioeconomic status.	13

D&B score, Downs and Black score,^14^ which evaluates the quality of articles and ranges from 0–20 points.

**Table 2:** Synthesis of the selected studies published from 2010 onwards.

Authors	Sample/nationality	Sleep variables	Socioeconomic variables	Results	D&B score
Siomos et al. (2010)^7^	2195 individuals from Greece aged 13–18 years	Sleep latency, nighttime awakenings, sleep duration, welfare and daytime sleepiness	Parental schooling and family financial status	Young individuals who perceive higher family financial status were less likely to suffer from insomnia. There was no significant association between parental level of schooling and insomnia complaints.	9
Moore et al. (2011)^19^	247 individuals from the USA aged 13–16 years	Sleep duration	Ethnicity, income, parental level of schooling and neighborhood characteristics	Ethnicity was associated with sleep duration, indicating that adolescents belonging to majority ethnic groups had 19.91 min more sleep than the minority ones. Similarly, adolescents living in less problematic neighborhoods slept on average more than the others. The correlations showed that sleep duration was positively and significantly correlated with parental income.	15
Marco et al. (2011)^20^	155 individuals from the USA with a mean age of 12.6 (0.6) years	Sleep duration and sleep pattern consistency	Income, parental level of schooling and family environment and neighborhood	Young individuals of low socioeconomic status had lower sleep duration and a later bedtime. Additionally, young individuals with poorer social and environmental indicators had less consistent and more irregular sleep pattern.	11
Bøe et al. (2012)^21^	5781 individuals from Norway aged 11–13 years	Difficulty initiating or maintaining sleep and sleep duration	Parental level of schooling and perception of family economic status	Poor socioeconomic status was associated with difficulties for the young individual to initiate and/or maintain sleep, while low sleep duration was associated only to the perceived family socioeconomic status as poor. Higher level of maternal education was associated with longer duration of adolescent sleep.	13
Fernando et al. (2013)^22^	1388 individuals from New Zealand aged 14–23 years	Sleep disorders, duration of sleep problems and drug use related to sleep	Ethnicity and economic profile of schools	No differences were observed regarding the assessed sleep disorders considering the economic profile of schools. Additionally, sleep disorders were similar between the ethnic groups investigated.	11
Jarrin et al. (2013)^23^	239 individuals from Canada aged 8–17 years	Sleep quality, disorders and duration, daytime sleepiness	Family income, parental education and social status	Adolescents with low social indicators had a poor sleep pattern. Nevertheless, the objective socioeconomic measures had greater explanatory power in childhood, whereas the subjective indicator was more relevant in adolescents.	13

D&B score, Downs and Black score,^14^ which evaluates the quality of articles and ranges from 0–20 points.

The most often investigated sleep variables were sleep duration, quality and disorders. Considering the socioeconomic variables, we observed several analysis parameters, such as schooling level of parents or guardians, income, ethnicity and socioeconomic status or level.

Of the six studies that analyzed adolescents from different ethnic backgrounds, differences were observed related to sleep quality and sleep disorders such as insomnia or hypersomnia, in three of them. In other studies, which compared sleep variables according to different ethnic groups, it was observed that prevalence of sleep disorders or poor quality of sleep was similar between the groups.

The researchers reported, in five reviewed studies, that the adolescents from low-income families or with more obvious indicators of poverty were more likely to develop sleep disorders, such as insomnia and difficulty initiating and/or maintaining sleep.

Sleep duration was associated with parental income and ethnicity, and three studies indicated a decline in sleep duration in young individuals of lower socioeconomic status when compared to those of higher socioeconomic status. In the only study carried out in Brazil, sleep duration tended to decrease with increasing socioeconomic status. Regarding the measures applied in the studies, two articles used an actimeter as the objective measure to evaluate the duration of sleep, whereas the other studies used subjective measures such as questionnaires and interviews.

Quality assessments of the selected studies are also described in Table 1 (D&B score). The median score, according to the proposal by Downs and Black,^14^ was 12.2 (minimum of nine and maximum of 15 points). The mean score of the articles was 12, with a standard deviation of 1.78 point. Overall, the methodological limitations of the selected articles were related to the descriptions of individuals, considering, for instance, the need for more detailed information on sample loss. It is worth mentioning that, of the 12 articles, none provided information on the statistical power of the tests used. Furthermore, the questions that best met the criteria proposed for quality analysis are related to the internal validity (bias), present in all the analyzed articles.

## Discussion

Socioeconomic level is a theoretical construct that aims to empirically classify individuals into social classes and strata. Although its definition is not unanimous, it has been traditionally based on family income, level of schooling and occupation.^24^ The social stratum of individuals and communities involves a complex construction of economic, social, environmental and behavioral components, which define and delineate opportunities for and barriers to development and also the higher or lower probability of having certain health conditions. Nevertheless, studies have shown possible associations between social aspects and sleep.^4^^,^ 15 ^,^ 22

In this sense, this systematic review aimed to analyze the association between socioeconomic status and sleep in adolescents, as this is an age group that is particularly vulnerable to sleep problems. The selected articles aimed to answer how sleep variables, such as sleep quality, time to wake up and go to sleep, sleep duration, daytime sleepiness, among others, are associated with socioeconomic indicators of the adolescents.

In general, there is an association between sleep variables and socioeconomic status. Jarrin, McGrath and Quon,^23^ for instance, explain that families with lower socioeconomic status have less organized houses, with more noise and less knowledge about sleep hygiene. Thus, the low social status could be a stressor and reduce the quality of sleep. Among the analyzed studies,^6^^,^ 20 ^,^ 21 this association seems to be a trend among adolescents from foreign samples. However, for Smaldone et al.,^18^ this outcome was not the same. In this study, non-Hispanic white adolescents with higher level of family education were approximately 30% more likely to have inadequate sleep patterns, when compared with other ethnic groups (non-Hispanic blacks, Hispanics and others). In the analyzed studies, associations between ethnicity and socioeconomic variables showed no agreement.^15^^-^ 17 ^,^ 19 ^,^ 22 ^,^ 25 One of the explanations, according to Roberts et al.,^17^would be that sleep problems are associated with the social status of a minority, not being associated with ethnicity. Another important point, according to some authors,^16^^,^ 17 is the fact that young individuals who belong to minority groups can express mental suffering in a negative way, especially among immigrants. These are more susceptible to ethnic prejudice and negative stereotypes and, therefore, more likely to internalize their minority status, which can affect their perceptions and behaviors.

The study by Marco et al.^20^ was the only one to carry out a discussion on the associations between sleep and socioeconomic levels, considering school and non-school days. In general, it was found that school hours, as well as housing and hygiene conditions modify the efficiency and duration of sleep, with the low-income adolescents showing worse sleep quality on schooldays and weekends. Nevertheless, housing conditions and neighborhood characteristics become more important on weekends, as on school days, the school schedule contributes to the adolescents’ sleep regulation.

In addition to quality, other sleep variables were associated in the selected studies, especially sleep disorders, such as insomnia,^15^^-^ 17hypersomnia,^15^ nocturnal awakenings,^7^^,^ 17 ^,^ 23 restless leg syndrome, sleepwalking, talking during sleep, bruxism and delayed sleep phase.^22^ Excessive daytime sleepiness is a possible indicator of increased need for sleep and is associated with decreased school performance, negatively influencing learning, social interaction and adolescent quality of life. Adolescence is associated with delayed sleep phase and the morning school hours, which lead to decreased sleep duration.^2^ Additionally, as verified in this review, excessive daytime sleepiness is associated with both biological and behavioral factors and with variables related to the environment where the adolescent lives.^7^^,^ 23

Based on the reading of the selected articles, it was observed that different indicators were used to determine the socioeconomic status of adolescents. In international studies, the questions on parental level of schooling and family income are the ones more often used. In Brazil, it is common to use the economic classification criterion of the Brazilian Association of Population Studies - ABEP. This is a questionnaire that takes into account the head of the family's educational level, the possession of consumer goods such as home appliances, electronics and automobiles. The sum of the number of items provides a score, which categorizes the family in relation to its economic status.^26^

It was verified that the main form of sleep assessment was through questionnaires, i.e., subjectively. Among these, we highlight questions related to sleep duration, obtained from the bedtime and wake up time reported by adolescents. Few studies used direct measures, such as the actimeter, for their analysis.^19^^,^ 20 This tool is characterized by being an objective measure, similar to a wristwatch, which contains a device that, based on the individual's movement, estimates sleeping and waking hours through electronic sensors.^19^Additionally, the actigraphy allows obtaining more precise information on the times with greater and lesser motor activity during the day and night as well as sleep latency and efficiency. In both studies using actigraphy, it was observed that young individuals with low socioeconomic status^20^and from ethnic minorities^19^ had shorter sleep duration.

There was a methodological limitation of the articles mentioned in this review when considering the checklist proposed by Downs and Black.^14^ The description of the statistical power of the statistical tests used in the studies was not shown in any of the articles, indicating that this information needs to be better understood and described by researchers.

The only study with a Brazilian sample was the one by Bernardo et al.,^4^ carried out with adolescents from São Paulo. This study identified trends that were opposite to the ones found in foreign samples, i.e., adolescents with higher socioeconomic status had worse indicators of sleep. Thus, it is necessary to develop other Brazilian studies to better support the educational proposals in health and sleep, considering the reality of developing countries.

## Final considerations

Based on the analysis of the articles selected for this systematic review, we observed a significant association between socioeconomic indicators and sleep of adolescents. In general, the low socioeconomic status is reflected in a worse subjective perception of sleep quality, shorter duration and greater daytime sleepiness.

Regarding ethnic factors, a more direct investigation is required with an epidemiological context to try to explain whether there is an association with sleep variables.

In spite of the strong influence of the socioeconomic context on the quantity and quality of sleep observed in the analyzed studies, the number of studies on sleep of adolescents from different socioeconomic levels in poor and developing countries is scarce. The only study with a Brazilian sample indicated associations that were contrary to those seen in the foreign studies, showing shorter duration of sleep among adolescents in higher socioeconomic classes.^4^

Considering that sleep can influence the behavioral and emotional states, physical and cognitive development, in addition to levels of attention and learning of adolescents or students in general, it is emphasized that bad sleep habits, initiated in childhood and adolescence, can persist into adulthood. Therefore, we need more studies on the associations between sleep and socioeconomic status in order to attain better planning of public and educational policies.
