# Supplementary material for: Sleep in adolescents of different socioeconomic status: a systematic review
Source: Rev Paul Pediatr. 2015 Oct-Dec;33(4):467–73. doi: 10.1016/j.rpped.2015.01.011 (PMC4685568; doi:10.1016/j.rpped.2015.01.011)
Supplement: Translation

# Translation: Sono em adolescentes de diferentes níveis socioeconômicos: revisão sistemática

**Authors:** Érico Pereira Gomes Felden, Carina Raffs Leite, Cleber Fernando Rebelatto, Rubian Diego Andrade, Thais Silva Beltrame

**Affiliations:** a Universidade do Estado de Santa Catarina (UDESC), Florianópolis, SC, Brasil

**Keywords:** Sono, Adolescentes, Nível socioeconômico

## Abstract

**Objetivo:**

Analisar as características do sono em adolescentes de diferentes níveis socioeconômicos.

**Fontes de dados:**

Foram analisados estudos encontrados nas bases de dados Medline/PubMed e SciELO que apresentassem resultados originais, sem restrições de idioma e de período, com associações entre variáveis de sono e indicadores socioeconômicos. A busca inicial teve como resultado 99 estudos. Diante dos critérios de inclusão, exclusão e após a leitura dos textos completos, 12 artigos apresentaram em seus desfechos associações entre as variáveis de sono (distúrbios, duração e qualidade) com os parâmetros socioeconômicos (etnia, renda e classe social).

**Síntese dos dados:**

Os estudos que relacionam o sono com variáveis socioeconômicas são recentes e datados a partir de 2000. Metade das pesquisas selecionadas foi feita com jovens americanos e apenas uma com adolescentes brasileiros. Com relação às diferenças étnicas, os estudos não apresentam conclusões uniformes. As principais associações foram com a renda familiar e nível de escolaridade dos pais e evidenciaou-se uma tendência a jovens pobres e com *status* social mais baixo manifestarem baixa duração e má qualidade do sono.

**Conclusões:**

Constatou-se associação entre os indicadores socioeconômicos e o sono dos adolescentes. O baixo *status* socioeconômico refletiu-se numa pior percepção subjetiva da qualidade do sono, menor duração e maior sonolência diurna. Considerando a importância do sono para o desenvolvimento físico, cognitivo e na aprendizagem dos jovens, o número de pesquisas ainda é escasso. Sugerem-se mais investigações sobre o sono em diferentes realidades da população brasileira.

## Introdução

As pessoas passam por grandes mudanças no decorrer das suas vidas, tanto na forma física quanto no comportamento. Na adolescência, em especial, é possível observar importantes mudanças na expressão do ciclo vigília/sono, incluindo um atraso na fase de sono, caracterizado por horários de dormir e acordar mais tardios.^1^^,^ 2 Essa tendência biológica dos adolescentes pode ser acentuada por comportamentos como o uso de computadores, jogos e televisão durante a noite. Além disso, questões ambientais, como os compromissos sociais no início da manhã, aumentam a prevalência de baixa duração do sono nessa população.^3^

O estudo de Bernardo et al.^4^ identificou prevalência de 39% de adolescentes com baixa duração do sono em São Paulo. Já Perez-Chada et al.^5^ observaram que 49% dos adolescentes argentinos investigados apresentavam baixa duração do sono. Problemas com o sono vêm sendo associados a diversos desfechos em saúde, como problemas de desenvolvimento cognitive,^6^distúrbios psíquicos,^7^ problemas metabólicos e de excesso de peso,^8^^,^ 9 bem como com maior percepção de estresse.^10^

Além das questões biológicas, o ambiente parece influenciar de forma decisiva a expressão do ciclo vigília/sono. Nesse contexto, a literatura aponta que o nível socioeconômico é uma das variáveis sociais mais relevantes para o entendimento das questões de saúde.^11^^-^ 13 Já com relação ao sono, pode-se perceber uma carência de estudos. Essa associação é pouco explorada, especialmente em se tratando de estudos com adolescentes. No entanto, entende-se que a compreensão das associações e dos nexos causais entre sono e nível socioeconômico seja fundamental para o entendimento do sono do adolescente e para mediar proposta de educação em saúde.

Diante do exposto, considerando-se a importância dos estudos que investiguem a relação entre o sono e o nível socioeconômico para o planejamento de ações em saúde pública e a escassez de trabalhos que sintetizem a literatura sobre essa temática, o presente estudo teve como objetivo fazer uma revisão sistemática para analisar a relação entre as caraterísticas do sono em adolescentes de diferentes níveis socioeconômicos.

## Método

A revisão sistemática de literatura foi feita com as bases de dados SciELO e Medline/PubMed, sem recorte de período ou exclusão de idioma. Foram usados na busca os termos "sono" (*sleep*) e "nível socioeconomic" (*socioeconomic status*) em cruzamento com o termo "adolescents" (*adolescents*). Além disso, a busca foi ampliada pela pesquisa de estudos relevantes nas referências bibliográficas dos artigos encontrados na busca inicial. A primeira busca apresentou 99 estudos, conforme descrito na Figura 1.

**Figura 1:** Fluxograma de processo de seleção de artigos para a revisão.

A partir da busca inicial, os artigos selecionados para análise deveriam preencher os seguintes critérios de inclusão: a) artigos originais com resultados de variáveis de sono (duração e qualidade do sono, eficiência do sono e distúrbios leves de sono como insônia); b) com amostras de adolescentes e c) que apresentassem medidas de associação e/ou diferenças entre as variáveis de sono e indicadores socioeconômicos. Foram excluídos aqueles artigos que tratassem de pesquisas em amostras com patologias específicas, como deficiência mental e cardiopatias.

Considerando os critérios de inclusão e exclusão citados, a partir da leitura dos títulos e resumos, 85 artigos foram excluídos, por não estratificar os adolescentes das amostras de crianças e adultos ou não envolver os adolescentes na população investigada e/ou por fazer análises somente em populações com doenças crônicas não transmissíveis e/ou com distúrbios mais graves de sono. Além desses, a maior causa de exclusão foi o fato de alguns artigos apresentarem as variáveis de sono e de nível socioeconômico como independentes e não mostrarem associações ou diferenças entre elas. Sendo assim, foram selecionados 14 estudos para a leitura integral. Após esse processo, foram feitos leituras, fichamento dos textos completos e discussão em grupo e selecionados nove artigos. Além disso, foram incluídos mais três artigos encontrados nas referências e se chegou a 12 artigos para a análise final. Os fichamentos foram feitos, de forma independente, por quatro pesquisadores e os resultados e as análises discutidos em grupo.

Fez-se a avaliação da qualidade dos artigos, com o uso da proposta elaborada por Downs e Black^14^ composta por 27 questões, que estimam a comunicação, a validade externa, a validade interna (viés e confusão) e o poder estatístico. Essa avaliação foi feita por dois autores. Nos casos de dúvidas, um terceiro avaliador foi consultado para decisão final. Para o presente estudo, as questões 8, 13, 14, 15, 17, 19, 20, 21, 22, 23, 24 e 26 foram excluídas, pois os estudos de intervenção não foram incluídos na revisão. Foram, dessa forma, analisadas 15 questões. Segundo a proposta de avaliação de qualidade, as questões receberam pontuação zero (0) ou um (1), exceto a questão 5, que variou de zero (0) a dois (2) pontos. Além disso, a questão 27, que analisa o poder estatístico, poderia variar de zero (0) a cinco (5). Assim, um artigo poderia obter a pontuação máxima de 20 pontos. Tendo em vista o número baixo de artigos selecionados, essa análise teve como objetivo discutir fatores relacionados à qualidade dos artigos e não se constituiu em critério de exclusão.

## Resultados

Nas Tabelas 1 e 2 são apresentadas as informações gerais sobre os 12 estudos incluídos nesta revisão, de acordo com a data da publicação (2000 a 2009 na Tabela 1 e de 2010 adiante na Tabela 2). As amostras provêm de diferentes nacionalidades, seis estadunidenses, uma neozelandesa, uma australiana, uma grega, uma norueguesa, uma canadense e uma brasileira. As publicações aconteceram de 2000 a 2013, 11 delas com delineamento transversal e apenas uma longitudinal.

**Tabela 1:** Síntese dos estudos selecionados publicados entre 2000 e 2009

Autores	Amostra/nacionalidade	Variáveis de sono	Variáveis socioeconômicas	Resultados	Escore D&B
Roberts et al. (2000)^15^	5.423 jovens americanos de 10 a 17 anos	Insônia e hipersonia	Etnia e percepção de *status*socioeconômico	Os adolescentes de diferentes origens étnicas apresentaram diferenças relacionadas a insônia e hipersonia. A insônia foi mais frequente nos grupos de origem africana e hispânica. Já os adolescentes de origem chinesa apresentaram menos risco de problemas com o sono	13
Roberts et al. (2004)^16^	5.118 jovens americanos de 13 a 18 anos	Insônia, duração do sono, qualidade do sono e sono reparador	Etnia e padrão de vida da família	O estudo apresentou diferenças significativas entre as etnias com relação à insônia e qualidade do sono. A insônia foi mais presente nos adolescentes pobres. As associações entre insônia e etnia não foram significativas após ajustes por variáveis de confusão. Aqueles adolescentes classificados como “pobres” apresentaram 4,5 vezes mais probabilidade de relatar a insônia.	12
Roberts et al. (2006)^17^	4.175 jovens americanos de 11 a 17 anos e cuidadores adultos	Problemas para adormecer, despertares noturnos, insônia e sono não reparador	Etnia, renda familiar e nível de escolaridade dos cuidadores	Os jovens de baixa renda familiar tiveram maiores riscos de desenvolver distúrbios do sono. Jovens de descendência europeia apresentaram maiores chances de ter dificuldades para dormir, de ter problemas com sono (como acordar no meio da noite) e dificuldade para dormir do que os com descendência africana. Já os jovens de origem hispânica apresentaram menor prevalência de problemas para dormir do que os jovens de descendência europeia.	11
Smaldone et al. (2007)^18^	68.418 jovens americanos de 6 a 17 anos	Qualidade do sono	Etnia, escolaridade dos pais, renda e cor da pele	Adolescentes brancos não hispânicos, com maior nível de escolaridade familiar, apresentaram cerca de 30% a mais de chance de ter padrões de sono inadequados. Porém, verificou‐se que os adolescentes com menor renda familiar tinham 50% a menos de chance de apresentar padrões de sono inadequado, tendo como referência os adolescentes com maior renda familiar.	14
Dollman et al. (2007)^6^	900 jovens australianos de 10 a 15 anos (390 em 1985 e 510 em 2004)	Duração do sono, horários de dormir e acordar	Índice socioeconômico australiano	Na comparação entre as duas avaliações houve redução mais acentuada na duração do sono nos jovens com nível socioeconômico mais baixo (44 min) em relação aqueles com *status* socioeconômico mais elevado.	9
Bernardo et al. (2009)^4^	863 jovens brasileiros de 10 a 19 anos	Duração do sono e distúrbios de sono	Classes sociais	A duração do sono apresentou tendência de diminuição com o aumento do nível socioeconômico	13

Escore D&B, Escore de Downs & Black^14^ que avalia a qualidade dos artigos e varia de 0‐20 pontos.

**Tabela 2:** Síntese dos estudos selecionados publicados de 2010 em diante

Autores	Amostra/nacionalidade	Variáveis de sono	Variáveis socioeconômicas	Resultados	Escore D&B
Siomos et al. (2010)^7^	2195 jovens gregos de 13 a 18 anos	Latência do sono, despertares noturnos, duração do sono, bem‐estar e sonolência diurna	Escolaridade dos pais e situação financeira familiar	Jovens com percepção de situação financeira familiar mais elevada apresentaram menor probabilidade de sofrerem insônia. Não houve relação significativa entre o nível de escolaridade dos pais e queixas de insônia.	9
Moore et al. (2011)^19^	247 jovens americanos de 13 a 16 anos	Duração do sono	Etnia, renda, nível de escolaridade dos pais e características do bairro	Etnia esteve associada com a duração do sono, indicando que aqueles adolescentes de etnia maioritária tinham 19,91 min a mais do que os de minoria. Do mesmo modo, os adolescentes que vivem em bairros menos problemáticos dormiram, em média, mais do que os outros. As correlações mostraram que a duração do sono foi positiva e significativamente correlacionada com a renda dos pais.	15
Marco et al. (2011)^20^	155 jovens americanos com média de idade de 12,6 (0,6) anos	Duração do sono e consistência no padrão de sono	Renda, escolaridade dos pais e ambiente familiar e da vizinhança	Jovens com baixo nível socioeconômico apresentaram menor duração do sono e horários mais tardios de dormir. Além disso, jovens com indicadores sociais e de ambiente mais pobres tinham sono menos consistente e com maior irregularidade.	11
Bøe et al. (2012)^21^	5.781 jovens noruegueses de 11 a 13 anos	Dificuldade em iniciar ou manter o sono e duração do sono	Escolaridade dos pais e percepção da situação economia familiar	A economia familiar pobre esteve associada com dificuldades do jovem em iniciar e/ou manter o sono. Enquanto que a baixa duração de sono esteve associada apenas a economia familiar percebida como pobre. Maior nível de escolaridade da mãe foi associado com maior duração de sono do adolescente.	13
Fernando et al. (2013)^22^	1.388 jovens neozelandeses de 14 a 23 anos	Distúrbios de sono, duração de problemas de sono e uso de medicamentos relacionados com o sono	Etnia e perfil econômico das escolas	Não foram observadas diferenças nas questões de sono avaliadas considerando o perfil econômico das escolas. Além disso, os distúrbios de sono foram semelhantes entre as etnias investigadas.	11
Jarrin et al. (2013)^23^	239 jovens canadenses de 8 a 17 anos	Qualidade, distúrbios e duração do sono, sonolência diurna	Renda familiar, escolaridade dos pais e *status* social	Adolescentes com baixos indicadores sociais apresentaram sono ruim. Apesar disso, as medidas socioeconômicas objetivas tiveram maior poder explicativo na infância, ao passo que o indicador subjetivo ganhou relevância nos adolescentes.	13

Escore D&B, Escore de Downs & Black^14^ que avalia a qualidade dos artigos e varia de 0‐20 pontos.

As variáveis do sono mais investigadas foram a duração, a qualidade e os distúrbios. Considerando as variáveis socioeconômicas, foi possível observar inúmeros parâmetros de análise, como a escolaridade dos pais ou cuidadores, renda, etnia e nível ou *status* socioeconômico.

Dos seis estudos que analisaram adolescentes de diferentes origens étnicas, foram observadas diferenças relacionadas à qualidade do sono e de distúrbios do sono, como insônia ou hipersonia, em três deles. Nos demais estudos, que compararam variáveis de sono de acordo com diferentes etnias, foram observadas prevalências de distúrbios do sono ou de baixa qualidade de sono semelhantes entre os grupos.

Os pesquisadores relataram em cinco estudos revisados que os jovens de baixa renda familiar, ou com indicadores mais evidentes de pobreza, apresentaram maiores chances de desenvolver distúrbios do sono, como a insônia e dificuldades de iniciar e/ou manter o sono.

A duração do sono foi relacionada com a renda dos pais e etnia. Três estudos apontaram para uma redução na duração do sono nos jovens com nível socioeconômico mais baixo em relação aqueles com melhor *status*socioeconômico. Já no único estudo brasileiro encontrado, a duração do sono apresentou tendência à diminuição com o aumento do nível socioeconômico. Com relação às medidas aplicadas nos estudos, dois artigos usaram o actímetro como medida objetiva para avaliar a duração do sono e os demais estudos usaram medidas subjetivas, como questionários e entrevistas.

As avaliações de qualidade dos estudos selecionados também estão descritas na Tabela 1 (Escore D&B). A mediana da pontuação, segundo a proposta de Downs & Black,^14^ foi de 12,2 (mínimo de nove e máximo de 15 pontos). A média da pontuação dos artigos foi de 12, com um desvio padrão de 1,78 ponto. De maneira geral, as limitações metodológicas dos artigos selecionados foram relativas às descrições dos indivíduos, considerando, por exemplo, a necessidade da apresentação mais detalhada das perdas amostrais. Cabe destacar que, dos 12 artigos, nenhum apresentou informações referentes ao poder estatístico dos testes usados. Além disso, as questões que melhor atenderam aos critérios propostos para análise da qualidade dizem respeito à validade interna (viés), presente em todos os artigos analisados.

## Discussão

O nível socioeconômico constitui-se um constructo teórico que visa a classificar, empiricamente, os indivíduos em classes ou estratos sociais. Embora não haja uma unanimidade em sua definição, tradicionalmente esse se baseia na renda familiar, no nível de escolaridade e na ocupação.^24^O estrato social de pessoas e comunidades envolve uma complexa construção de componentes econômicos, sociais, ambientais e comportamentais, que definem e delimitam oportunidades e barreiras para o desenvolvimento e também a maior ou menor probabilidade de se ter determinada situação de saúde. Não obstante, estudos apontam possíveis relações entre aspectos sociais e o sono.^4^^,^ 15 ^,^ 22

Nesse sentido, esta revisão sistemática procurou analisar a relação entre o nível socioeconômico e o sono de adolescentes, já que se trata de uma faixa etária especialmente vulnerável para apresentar problemas de sono. Os artigos selecionados procuraram responder de que forma as variáveis do sono, tais como qualidade do sono, horário para acordar e dormir, duração do sono, sonolência diurna, entre outras, estão associadas a indicadores socioeconômicos dos adolescentes.

De forma geral, há uma relação entre as variáveis do sono e *status* socioeconômico. Jarrin, McGrath e Quon,^23^ por exemplo, explicam que famílias com menor nível socioeconômico teriam casas menos organizadas, com mais barulho e menor conhecimento sobre higiene do sono. Com isso, o baixo *status* social poderia ser um agente estressor e redutor da qualidade do sono. Dentre os estudos encontrados,^6^^,^ 20 ^,^ 21 essa relação aparece como uma tendência entre os adolescentes de amostras estrangeiras. Porém, para Smaldone et al.,^18^ esse desfecho não foi o mesmo. Nesse estudo, os adolescentes brancos não hispânicos e com maior nível de escolaridade familiar tinham cerca de 30% a mais de chance de ter padrões de sono inadequados, quando comparados com outras etnias (negros não hispânicos, hispânicos e outros). Nos estudos analisados, as relações entre etnias e as variáveis socioeconômicas não entraram em consonância.^15^^-^ 17 ^,^ 19 ^,^ 22 ^,^ 25 Uma das explicações, segundo Roberts et al.,^17^seria que os problemas do sono estariam relacionados à condição social de minoria, e não à origem étnica. Outro ponto a ser destacado, segundo alguns autores,^16^^,^ 17 é o fato de jovens pertencentes ao grupo das minorias poderem expressar de forma negativa o sofrimento psíquico, especialmente entre os imigrantes. Esses são mais suscetíveis a preconceitos étnicos e a estereótipos negativos e, portanto, mais propensos a internalizar a sua condição de minoria, o que pode afetar suas percepções e seus comportamentos.

O estudo de Marco et al.^20^ foi o único a fazer uma discussão das associações entre sono e nível socioeconômico, considerando os dias com e sem aula. De forma geral, foi verificado que tanto os horários escolares como as condições de moradia e higiene modificam a eficiência e a duração do sono. Os adolescentes de baixa renda apresentam piores condições de sono nos dias com aula e fins de semana. Apesar disso, as condições de moradia e características da vizinhança ganham maior importância nos fins de semana, já que nos dias com aula os horários escolares contribuem para a regulação do sono dos adolescentes.

Além da qualidade, outras variáveis do sono foram relacionadas nos estudos selecionados, com destaque para os distúrbios do sono, tais como: a insônia,^15^^-^ 17 hipersonia,^15^ despertares noturnos,^7^^,^ 17 ^,^ 23 síndrome das pernas inquietas, sonambulismo, fala durante o sono, bruxismo, atraso da fase do sono.^22^ A sonolência diurna excessiva é um possível indicador de maior necessidade de sono e está associada à diminuição do aproveitamento escolar e influência negativamente a aprendizagem, a interação social e a qualidade de vida do adolescente. A adolescência está ligada ao atraso da fase de sono e aos horários escolares matutinos que levam à diminuição da duração do sono.^2^ Ainda, conforme verificado na presente revisão, a sonolência diurna excessiva está associada tanto com fatores biológicos e comportamentais como com variáveis relacionadas ao ambiente no qual o adolescente está inserido.^7^^,^ 23

A partir da leitura das pesquisas selecionadas verificou-se que diferentes indicadores são usados para determinar o nível socioeconômico dos adolescentes. Nas pesquisas internacionais, as questões de escolaridade dos pais e renda familiar são usadas com maior frequência. No Brasil, é comum o uso do critério de classificação econômica da Associação Brasileira de Estudos Populacionais (Abep). Trata-se de um questionário que leva em consideração, além de questões de escolaridade do chefe da família, a posse de bens de consumo, como eletrodomésticos, eletroeletrônicos e automóveis. A soma do número de itens fornece uma pontuação, que categoriza a família em relação a sua classe econômica.^26^

Verificou-se que a principal forma de avaliação do sono foi por meio de questionários, ou seja, de forma subjetiva. Entre essas, destacam-se questões relativas à duração do sono, obtidas a partir da hora de dormir e acordar relatadas pelos adolescentes. Poucos estudos usaram medidas diretas, como o actímetro, para as análises.^19^^,^ 20 Esse instrumento caracteriza-se por ser uma medida objetiva, semelhante a um relógio de pulso, que contém um dispositivo que, baseado no movimento do indivíduo, estima a hora de dormir e hora de acordar, por meio de sensores eletrônicos.^19^ Além disso, com a actimetria é possível obter informações mais precisas sobre os momentos com maior e menor atividade motora durante o dia e a noite, bem como a latência e a eficiência do sono. Em ambos os estudos que usaram a actimetria foi observado que os jovens com nível socioeconômico baixo^20^ e de minoria étnica^19^apresentaram menor duração do sono.

Houve limitação metodológica dos artigos citados nesta revisão ao se considerar o *check-list* proposto por Downs & Black.^14^ A descrição do poder estatístico dos testes usados nos estudos não foi apresentada em qualquer dos artigos. Isso indica que essa informação precisa ser mais bem compreendida e descrita pelos pesquisadores.

O único estudo com amostra brasileira listado foi o de Bernardo et al.^4^ com adolescentes paulistas. Esse estudo identificou tendências contrárias aos estudos com amostras estrangeiras, ou seja, os adolescentes com melhor nível socioeconômico apresentaram piores indicadores de sono. Assim, faz-se necessário o desenvolvimento de outros estudos com amostras brasileiras para melhor fundamentar as propostas de educação em saúde e sono, considerando a realidade dos países em desenvolvimento.

## Considerações finais

A partir das análises dos artigos selecionados para esta revisão sistemática, constatou-se haver relação significativa entre os indicadores socioeconômicos e o sono dos adolescentes. De forma geral, o baixo *status*socioeconômico reflete-se numa pior percepção subjetiva da qualidade de sono, menor duração e maior sonolência diurna.

Com relação aos fatores étnicos, necessita-se de uma investigação mais direta e de âmbito epidemiológico, para tentar explicar se há alguma relação com as variáveis do sono.

Apesar da forte influência do contexto socioeconômico na quantidade e na qualidade do sono constatada nos estudos analisados, o número de pesquisas sobre o sono de adolescentes de diferentes níveis socioeconômicos em países pobres e em desenvolvimento é escasso. O único estudo com amostra brasileira indicou associações contrárias àquelas apresentadas nos estudos estrangeiros e mostrou menor duração do sono em adolescentes de classes mais altas.^4^

Considerando que o sono pode influenciar o estado comportamental e emocional, o desenvolvimento físico e cognitivo, além dos níveis de atenção e aprendizagem dos adolescentes, ou dos estudantes em geral, destaca-se que hábitos ruins de sono, iniciados na infância e na adolescência, podem acompanhar a pessoa na vida adulta. Portanto, são necessárias mais investigações sobre as relações entre o sono e o nível socioeconômico, com vistas a um melhor planejamento de políticas públicas e educacionais.
